# Supplementary material for: A methodological assessment of randomization integrity in alteplase for acute ischemic stroke individual patient data meta-analyses
Source: PLoS One. 2025 Mar 19;20(3):e0315342. doi: 10.1371/journal.pone.0315342 (PMC11922233; doi:10.1371/journal.pone.0315342)
Supplement: S1 Table — (DOCX) [file pone.0315342.s001.docx]

| **Signaling Question** | **Response** | **Justification from Trial Publication or Product Licensing Application** | **Additional Justification for Response** |
| --- | --- | --- | --- |
| Was the allocation sequence random? | Probably Yes | “A permuted-block design with blocks of various sizes was used for randomization, with patients stratified according to clinical center and time from the onset of stroke to the start of treatment (0-90 or 91-180 minutes).” [13] | No information on method used for random sequence generation. |
| Was the allocation sequence concealed until participants were enrolled and assigned to interventions? | Probably No | “The randomization process was decentralized.” [47]  “The ID number was random, not numerically sequential.” [46] | Randomization was done at treatment centers (N=39) but required coordination by clinical centers (N=8) and a coordinating center. Sequence ID numbers also employed for reuse of patient ID numbers. |
|  |  | “There are 16 patients of the total 624 reported as having been unblinded during the study. This includes 12 patients in the Activase group, and 4 in the placebo group.” [47] | Manila envelopes attached to study drugs were intended for emergent unblinding. Reasons for unblinding available for 8/16 participants. |
|  |  | “Blinding was incorporated into the studies by using blind labeled vials and identical administration regiments for the treatment arms.” [47] | Contents of matched placebo used to generate foaming reaction unreported. |
| Did baseline differences between intervention groups suggest a problem with the randomization process? | Yes | N/A | 4 baseline imbalances identified between groups prognostically favoring alteplase in the 91-180 minute stratum were consistent with a larger treatment effect compared to the 0-90 minute stratum. [15] |
| **Risk of Bias** | **High Risk of Bias** |  |  |
