# Supplementary material for: A methodological assessment of randomization integrity in alteplase for acute ischemic stroke individual patient data meta-analyses
Source: PLoS One. 2025 Mar 19;20(3):e0315342. doi: 10.1371/journal.pone.0315342 (PMC11922233; doi:10.1371/journal.pone.0315342)
Supplement: S2 Table — (DOCX) [file pone.0315342.s002.docx]

| **Signaling Question** | **Response** | **Justification from Trial Publication or Product Licensing Application** | **Additional Justification for Response** |
| --- | --- | --- | --- |
| Was the allocation sequence random? | Probably Yes | “The patients were randomized following a central code using a blocked randomization, stratified by clinical center.” | No information on method used for random sequence generation. |
| Was the allocation sequence concealed until participants were enrolled and assigned to interventions? | Probably Yes | “The study used an interactive voice system for randomization and drug supply management. No one at local site was aware of patient group assignment.” | N/A |
|  |  | “The study drug consisted of white lyophilized powder, indistinguishable between groups, that was reconstituted with sterile water.” | Contents of matched placebo used to generate foaming reaction unreported. |
| Did baseline differences between intervention groups suggest a problem with the randomization process? | No | N/A | Baseline difference in diabetes status (p=.03) in ATLANTIS B with slightly more participants with diabetes in the alteplase arm (25% vs. 18%). |
| **Risk of Bias** | **Low**  **Risk of Bias** |  |  |
