# Supplementary material for: A methodological assessment of randomization integrity in alteplase for acute ischemic stroke individual patient data meta-analyses
Source: PLoS One. 2025 Mar 19;20(3):e0315342. doi: 10.1371/journal.pone.0315342 (PMC11922233; doi:10.1371/journal.pone.0315342)
Supplement: S3 Table — (DOCX) [file pone.0315342.s003.docx]

| **Signaling Question** | **Response** | **Justification from Trial Publication or Product Licensing Application** | **Remarks** |
| --- | --- | --- | --- |
| Was the allocation sequence random? | Yes | “A computer-generated randomization procedure in blocks of four was used, with each centre allocated at least one block of the treatment groups at 0–3 h and 3–6 h to ensure a stratified distribution.” | N/A |
| Was the allocation sequence concealed until participants were enrolled and assigned to interventions? | Probably Yes | “The randomization schedule was known only to the Clinical Trial Support Unit at Boehringer Ingelheim and to one member of the External Safety Committee. At each centre, eligible patients were randomly assigned treatment at baseline by means of sequential patient numbers.”  “Treatment allocation was concealed from all investigators, but in emergencies, investigators had access to sealed opaque envelopes containing treatment allocation.” | Unknown if sequentially numbered drug containers, sequentially numbered envelopes, or other used.  Reasons for unblinding unreported. |
|  |  | “Alteplase (Boehringer Ingelheim, Ingelheim, Germany) and placebo were identical in appearance; they were sealed in vials containing 50 mg drug or placebo, reconstituted in 50 mL water for injection.” | Contents of matched placebo used to generate foaming reaction unreported. |
| Did baseline differences between intervention groups suggest a problem with the randomization process? | No | N/A | N/A |
| **Risk of Bias** | **Low Risk of Bias** |  |  |
