# Supplementary material for: A methodological assessment of randomization integrity in alteplase for acute ischemic stroke individual patient data meta-analyses
Source: PLoS One. 2025 Mar 19;20(3):e0315342. doi: 10.1371/journal.pone.0315342 (PMC11922233; doi:10.1371/journal.pone.0315342)
Supplement: S4 Table — Abbreviations: NIHSS, National Institute of Health Stroke Scale. (DOCX) [file pone.0315342.s004.docx]

| **Signaling Question** | **Response** | **Justification from Trial Publication or Product Licensing Application** | **Remarks** |
| --- | --- | --- | --- |
| Was the allocation sequence random? | Probably Yes | “Eligible patients were randomly assigned, in a 1:1 ratio, to receive .9 mg of alteplase (Actilyse, Boehringer Ingelheim) per kilogram, administered intravenously (with an upper limit of 90 mg), or placebo. | No information on method used for random sequence generation. |
| Was the allocation sequence concealed until participants were enrolled and assigned to interventions? | Probably Yes | “An interactive voice-randomization system was used, with randomization at centers performed in blocks of four to ensure a balanced distribution of group assignments at any time.” “The size of the blocks was withheld from the investigators to make sure that they were unaware of the treatment assignments”.  “Alteplase and matched placebo were reconstituted from a lyophilized powder in sterile water for injection.” | Contents of matched placebo used to generate foaming reaction unreported. |
| Did baseline differences between intervention groups suggest a problem with the randomization process? | Yes | N/A | Baseline imbalances in NIHSS score (p=.03) and previous stroke status (.003) prognostically favor alteplase. |
| **Risk of Bias** | **Some Concerns** |  |  |
