# Supplementary material for: A methodological assessment of randomization integrity in alteplase for acute ischemic stroke individual patient data meta-analyses
Source: PLoS One. 2025 Mar 19;20(3):e0315342. doi: 10.1371/journal.pone.0315342 (PMC11922233; doi:10.1371/journal.pone.0315342)
Supplement: S5 Table — (DOCX) [file pone.0315342.s005.docx]

| **Signaling Question** | **Response** | **Justification from Trial Publication or Product Licensing Application** | **Remarks** |
| --- | --- | --- | --- |
| Was the allocation sequence random? | Yes | “Numbers for treatment packs were generated by a computer programme for random allocation.” | N/A |
| Was the allocation sequence concealed until participants were enrolled and assigned to interventions? | Yes | “A block randomization design was used within each centre (four treatment packs per block). Treatment allocation was done by the Clinical Trials Pharmacy at the Royal Melbourne Hospital, Australia.” | N/A |
|  |  | “Patients were treated with the next sequentially numbered treatment pack, which contained either alteplase (0·9 mg/kg up to a maximum of 90 mg, 10% as bolus and the remainder over 1 h) or placebo in a double-blinded design.” | Contents of matched placebo used to generate foaming reaction unreported. |
| Did baseline differences between intervention groups suggest a problem with the randomization process? | No | N/A | N/A |
| **Risk of Bias** | **Low Risk of Bias** |  |  |
