# Supplementary material for: A methodological assessment of randomization integrity in alteplase for acute ischemic stroke individual patient data meta-analyses
Source: PLoS One. 2025 Mar 19;20(3):e0315342. doi: 10.1371/journal.pone.0315342 (PMC11922233; doi:10.1371/journal.pone.0315342)
Supplement: S6 Table — (DOCX) [file pone.0315342.s006.docx]

| **Signaling Question** | **Response** | **Justification from Trial Publication or Product Licensing Application** | **Remarks** |
| --- | --- | --- | --- |
| Was the allocation sequence random? | Yes | “Clinicians entered baseline data via a telephone voice- activated or a secure web-based randomisation system.” | N/A |
| Was the allocation sequence concealed until participants were enrolled and assigned to interventions? | Probably Yes | “Clinicians entered baseline data via a telephone voice- activated or a secure web-based randomization system.” | N/A |
|  |  | “The randomisation system informed local clinicians of the patients’ unique trial identification number, and the weight-adjusted dose of drug or placebo in the double- blinded phase, or of the weight-adjusted drug dose among those allocated thrombolysis in the open phase, to be given as a 10% bolus with the remainder by an infusion over 1 h.” | Most of trial open label. In blind phase, treatment packs used. |
| Did baseline differences between intervention groups suggest a problem with the randomization process? | No | N/A | N/A |
| **Risk of Bias** | **Low Risk of Bias** |  |  |
