# Supplementary material for: A methodological assessment of randomization integrity in alteplase for acute ischemic stroke individual patient data meta-analyses
Source: PLoS One. 2025 Mar 19;20(3):e0315342. doi: 10.1371/journal.pone.0315342 (PMC11922233; doi:10.1371/journal.pone.0315342)
Supplement: S7 Table — (DOCX) [file pone.0315342.s007.docx]

| **Trial** | **Age (yr)** | **t-Statistic** | **Weight (kg)** | **t-Statistic** | **NIHSS Score** | **t-Statistic** |
| --- | --- | --- | --- | --- | --- | --- |
| **NINDS rt-PA Stroke Study** | -2.04 | 2.19 | 3.78 | 2.69 | .84 | 1.47 |
| **ECASS-2** | NR | N/A | NR | N/A | NR | N/A |
| **ATLANTIS-A** | -2.0 | .95 | 1.00 | .31 | .10* | .09* |
| **ATLANTIS B** | -1.00 | 1.13 | 2.00 | 1.34 | .20* | .45* |
| **ECASS-3** | .70 | .86 | -.50 | .47 | .90 | 2.24 |
| **EPITHET** | -1.30 | .50 | NR | N/A | 0 | 0 |
| **IST-3** | -.41 | .93 | .35 | .65 | .-10 | .40 |

**S7 Table** : Mean differences in age, weight, and National Institute of Health Stroke Scale scores; and the associated t-statistic for all included trials. *Mean difference and t-statistic estimated from the reported p-value in the original trial for which only integer values were reported. In the ATLANTIS A RCT, a p-value of .58 was replicated from the reported p-value of .53. In the ATLANTIS B RCT, a p-value of .37 was replicated from the reported p-value of .36.
