# Supplementary material for: A methodological assessment of randomization integrity in alteplase for acute ischemic stroke individual patient data meta-analyses
Source: PLoS One. 2025 Mar 19;20(3):e0315342. doi: 10.1371/journal.pone.0315342 (PMC11922233; doi:10.1371/journal.pone.0315342)
Supplement: S8 Table — (DOCX) [file pone.0315342.s008.docx]

| **Type of selection bias** | **Description of selection bias** | **Risk factors for selection bias** | **Mechanism by which selection bias could have been introduced** | **Evidence from the trial data supporting the presence of selection bias** |
| --- | --- | --- | --- | --- |
| Second-order | Direct observation of allocation assignment due to failure of the allocation sequence concealment process. | Use of sealed envelopes, intended for emergent unblinding, were present on sites where participants were randomized. 8 envelopes were opened for undocumented reasons. | Ability to exclude eligible participants based on an investigator’s subjective interpretation of the baseline CT scan. A log of eligible, but excluded participants, was not kept. It was noted in the FDA PLA that “many” cases of patients were excluded after study material was prepared. | 1. Two centers had covariate imbalances in CT scan criteria favoring the alteplase group suggesting the suspected mechanism was plausible.      1. Highly improbable cross-over ratio (*p*=.0000052*).* 2. Imbalanced allocations in most strata despite using permuted blocks. 3. Violation of a pre-specified randomization rule at multiple centers regarding the ratio of participants enrolled in the two-time strata. 4. Multiple, unidirectional covariate imbalances in the 91–180-minute time strata favoring alteplase suggestive of imbalances due to biased randomization. |
| Third-order | Prediction, but not direct observation, of allocation assignment due to imperfect blinding leading to ineffective allocation sequence concealment under restrictive randomization procedures. | Use of stratified block randomization using random block sizes which are smaller on average.  Sources of inadvertent unblinding including potential lack of vial frothing in placebos, intracerebral hemorrhage, angioedema, and other adverse events that required unblinding per the study protocol. |  |  |
