# Supplementary figures and images for: A methodological assessment of randomization integrity in alteplase for acute ischemic stroke individual patient data meta-analyses
Source: PLoS One. 2025 Mar 19;20(3):e0315342. doi: 10.1371/journal.pone.0315342 (PMC11922233; doi:10.1371/journal.pone.0315342)

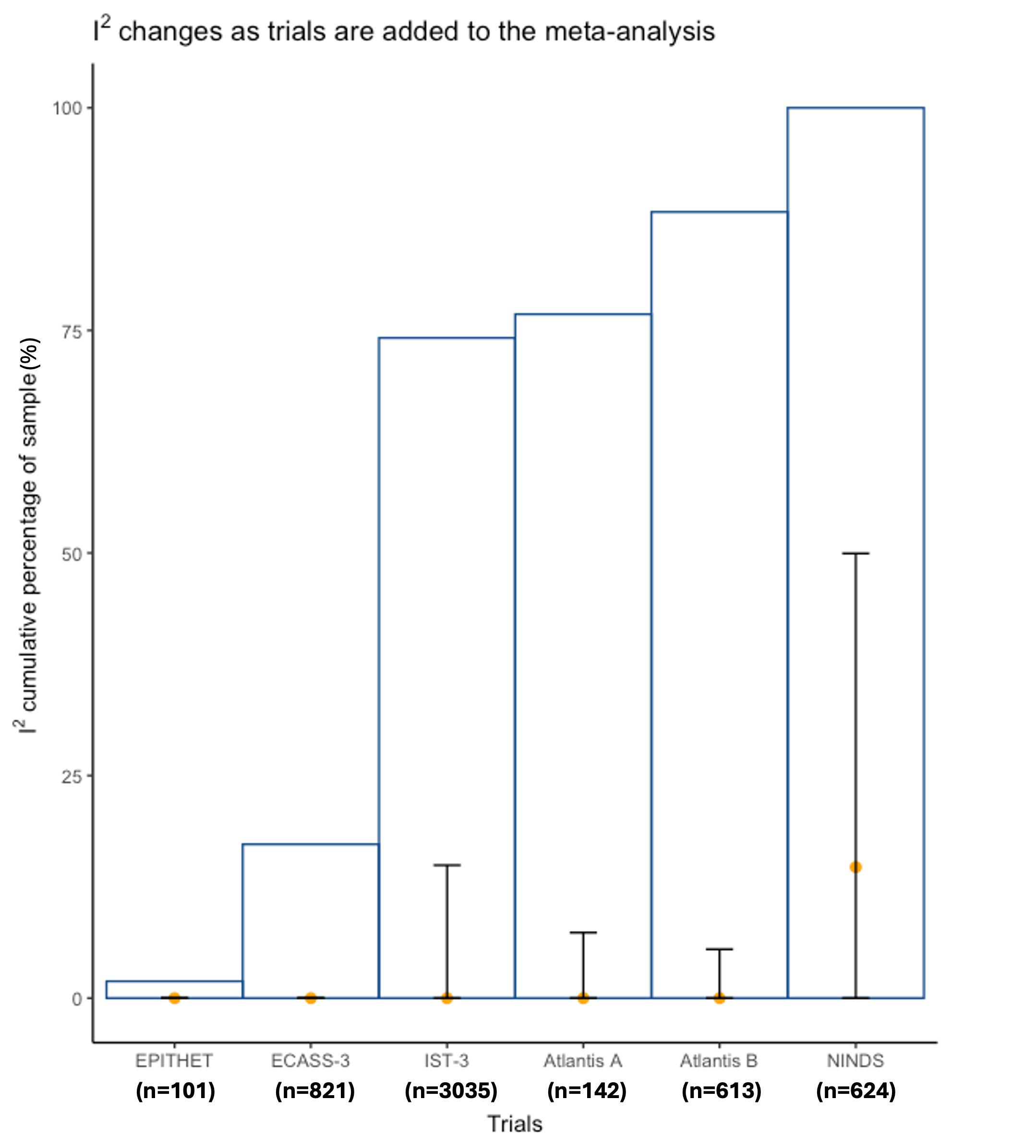

Supplement: S1 Fig — (TIF) [file pone.0315342.s009.tif]

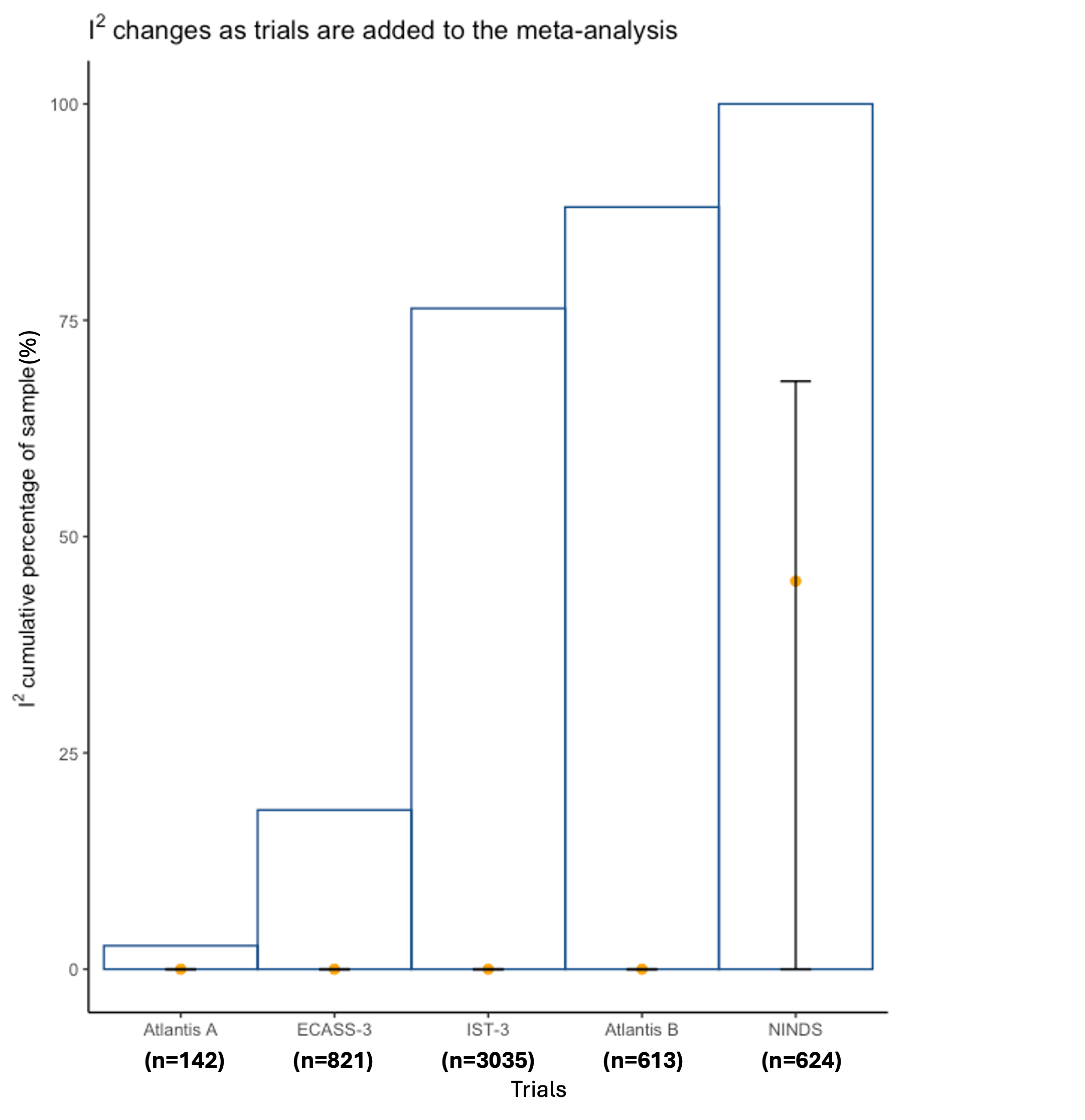

Supplement: S2 Fig — (TIF) [file pone.0315342.s010.tif]

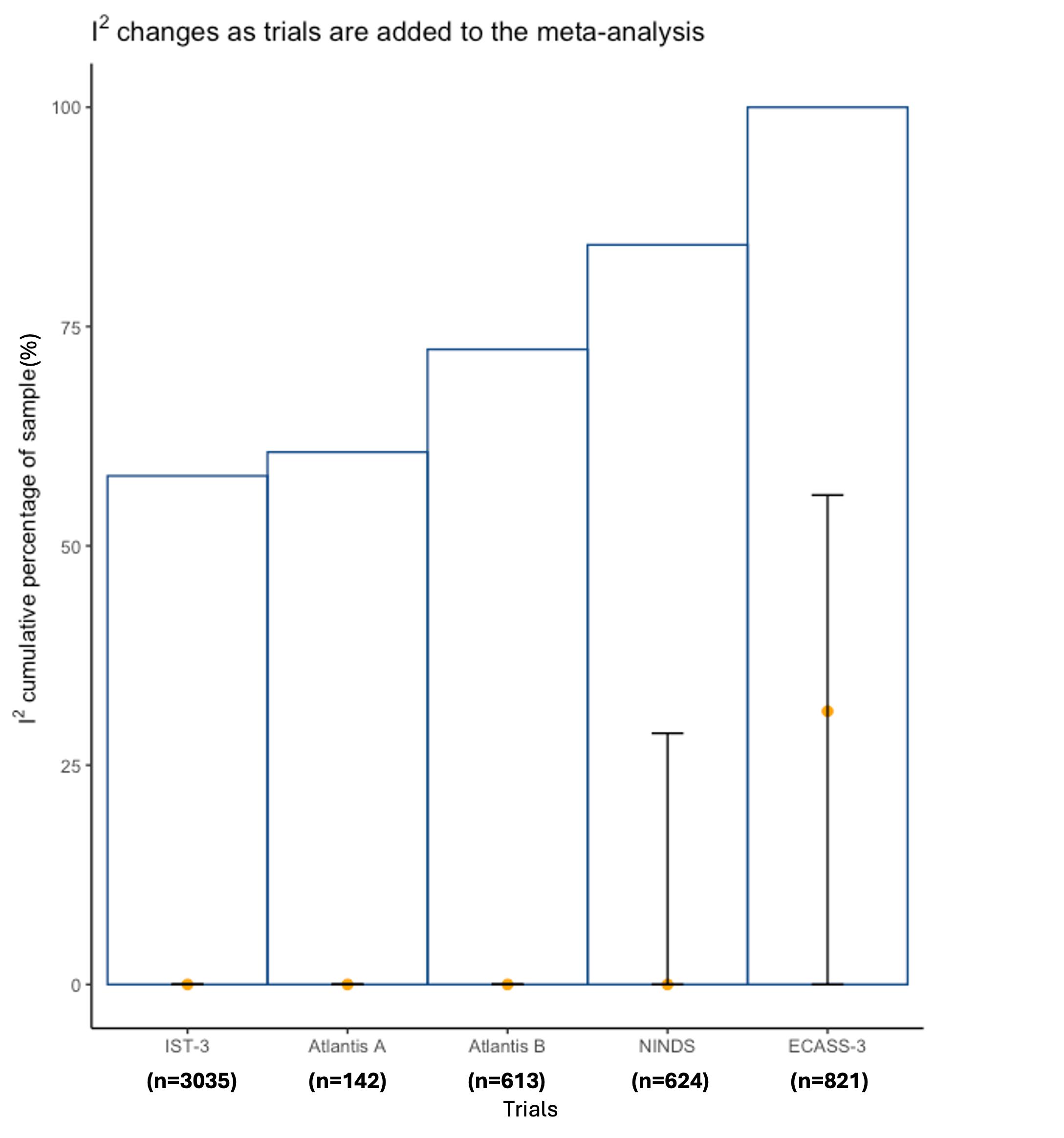

Supplement: S3 Fig — (TIF) [file pone.0315342.s011.tif]
